# miR-328-3p mediates the anti-tumor effect in osteosarcoma via directly targeting MMP-16

**DOI:** 10.1186/s12935-019-0829-7

**Published:** 2019-04-23

**Authors:** Jianhui Shi, Gang An, Ying Guan, Tianli Wei, Zhibin Peng, Min Liang, Yansong Wang

**Affiliations:** 1 Department of Spine Surgery, The First Affiliated Hospital of Harbin Medical University, Harbin, 150001 Heilongjiang Province China; 2 Department of Orthopaedics, Heilongjiang Provincial Hospital, No. 82, Zhongshan Road, Harbin, 150036 Heilongjiang China

**Keywords:** MiRNAs, Osteosarcoma, Proliferation, Migration, MMP-16

## Abstract

**Background:**

Increasing reports demonstrated that dysregulated expression of microRNAs (miRNAs) leads to the progression of various tumors. Previous studies revealed that miR-328-3p exhibited dysregulated expression in various types of tumors. However, its function and underlying mechanism in osteosarcoma (OS) are still unexplored.

**Methods:**

The expression of miR-328-3p in the tissues and OS cell lines was detected by qRT-PCR analysis. The effects of miR-328-3p in the proliferation were analyzed by MTT assay. The proliferation and apoptosis of OS cells were examined by colony formation assay and TUNEL staining respectively. The migration and tumor formation ability of OS cells were measured by wound healing assay and xenograft in vivo mice assay. Furthermore, the regulatory roles of miR-328-3p/MMP16 were determined by western blot and luciferase reporter assay.

**Results:**

The expression of miR-328-3p was significantly decreased in OS tissues and cell lines. Furthermore, overexpression of miR-328-3p inhibited the cell proliferation and migration, but promoted the apoptosis of OS cells in vitro. Moreover, the analysis in vivo showed that miR-328-3p effectively suppressed the formation of tumors. According to the results of western blot analysis and luciferase reporter assay, we identified matrix metalloproteinase-16 (MMP-16) acted as a direct target of miR-328-3p. Moreover, the expression level of MMP-16, which participates in the occurrence and development of many cancers, was negatively correlated with the miR-328-3p expression in OS cells.

**Conclusion:**

miR-328-3p inhibited the proliferation, migration but accelerated the apoptosis of OS by directly inhibiting MMP-16. And miR-328-3p/MMP-16 axis may be one of the mechanisms of OS development and a novel potential method for the treatment of OS in clinic.

## Background

Osteosarcoma (OS) is regarded as the most prevalent malignant bone cancer, which generally appears in the long bones of limbs as well as the growth plate nearby the metaphyseal [1, 2]. According to the reports, OS is usually observed in young adults and adolescents, and accounts for about 2.4% of all malignant tumors in children and exceeds about 20% of all primary bone cancers [3]. According to the reports, the morbidity of OS has reached approximately one to three cases annually per million all over the world [4]. Because of its extraordinarily high incidence, rapid progression, high malignance and great metastatic potential, the relative 5-year survival rate of patients with OS is less than 60% [5, 6]. In recent years, the treatments, including radiation therapy, adjuvant chemotherapy and surgery are used in the patients with OS. However, the prognosis of patients with OS remains poor and the survival rate of patients with OS has reached a plateau [7–9]. Therefore, investigating the molecular and molecular mechanisms of the development of OS and exploring new therapeutic approaches is really urgent.

MicroRNAs (miRNAs), a type of small, endogenous, single-stranded non-coding RNAs, are approximately 18 to 25 nucleotides in length [10, 11]. MiRNAs can bind directly to the 3′-untranslated region (3′-UTR) of target messenger RNAs (mRNAs) and negatively regulate the expression of specific genes [12, 13]. Therefore, miRNAs participate in both physiological and pathological conditions, including cell proliferation, differentiation, senescence and various diseases [14]. Accumulating evidence has revealed that abnormal expression of miRNAs are involved in various malignant tumors [15–17]. For example, it was reported that miR-302a plays a negative role in prostate cancer cell proliferation by inhibiting AKT [18]. Studies have also shown that the expression of miR-16 and miR-378 is up-regulated in osteoclast differentiation and they are related to bone metastasis burden [19]. In addition, miRNAs also serve as oncogenes or tumor suppressors with key roles in the development of OS [20, 21]. The crucial biological functions of miRNAs in OS have been gradually explored, but the underlying molecular and cellular mechanism has not yet been clarified.

In the present study, our data revealed miR-328-3p was observably decreased in the OS tissues and cell lines. A range of molecular biological methods were performed to detect the effect of miR-328-3p in the development and occurrence of OS, and to clarify the underlying molecular mechanisms. Our study showed that miR-328-3p might act as a tumor suppressor in OS and provided a theoretical basis for optimizing treatment strategies.

## Materials and methods

### Patient tissues

All the tumor tissues and corresponding adjacent tissues were obtained from the patients who were diagnosed of OS and received surgical resections at the Orthopedics of the First Affiliated Hospital of Harbin Medical University between October, 2017 and May, 2018. The OS tissues and corresponding adjacent tissues were collected in this study for estimation of miR-328-3p levels. After resections, the tissue samples were immediately frozen and stored in liquid nitrogen or at − 80 °C freezer. All the patients have not received any anticancer treatments, including radiotherapy or chemotherapy before surgery. The patients, who were diagnosed of active infections, HPV infections, chronic inflammatory diseases, were excluded from our study. All participants provided written informed consent before sample collection. The study was approved by the Ethics Committee of the First Affiliated Hospital of Harbin Medical University.

### Cell culture

Human OS cell lines (U2OS, Soas and MG63) and human osteoblasts (HOB) MC3T3 were purchased from the Zhong Qiao Xin Zhou Biotechnology, China. The cells were cultured in accordance to the instruction’s informations. All the cells were cultured in a 5% CO_2_ humidified incubator at 37 °C. The OS cell lines (MG-63 and Saos2) cultured in Dulbecco’s modified Eagle’s medium (DMEM) supplemented with 10% fetal bovine serum (Thermo, USA) and 1% penicillin/streptomycin (Beyotime, China). The OS cell lines (U2OS) were cultured in high glucose medium (Thermo, USA) supplemented with 10% FBS (Thermo, USA) and 1% penicillin/streptomycin (Beyotime, China). The HOB cells (MC3T3) were cultured in cultured in DMEM/F12 medium supplemented with 10% FBS and 1% penicillin/streptomycin (Beyotime, China). When the confluence of cells reached 80–90%, the cells were trypsinized by using trypsin (Beyotime, China) and were passaged to next passage.

### Cell transfection

MG-63 cells were plated in a 6-well plate or a 96-well plate. When the confluence of cells reached 40–50%, the cells were transiently transfected with the miR-328-3p (hsa-miR-328-3p) mimic, miR-328-3p inhibitor, mimic negative control (NC mimic), and inhibitor negative control (NC inhibitor). For miR-328-3p mimic, inhibitor, NC mimic and NC inhibitor were designed and synthesized by Genepharma (Shanghai, China). The sequence of hsa-miR-328-3p mimics was 5′-CUGGCCCUCUCUGCCCUUCCGU-3′ and 5′-GGAAGGGCAGAGAGGGCCAGUU-3′. The sequence of hsa-miR-328-3p inhibitor was 5′-ACGGAAGGGCAGAGAGGGCCAG-3′. The sequences of NC mimic were 5′-UUCUCCGAACGUGUCACGUTT-3′ and 5′-ACGUGAACAGUUCGGAGAATT-3′. The sequence of NC inhibitor was 5′-CAGUACUUUUGUGUAGUACAA. And all the sequences were transfected into OS cell line MG-63 by using X-treme reagent (Vazyme, China) according to the manufacturer’s protocol. Hsa-miR-328-3p mimic and NC mimic were transfected into the cells at a final concentration of 50 nM, while hsa-miR-328-3p inhibitor and NC inhibitor were transfected into the cells at a final concentration of 100 nM. The cells transfected with NC were used as control groups. After transfection of 24 h, the cells were used for further analysis and the efficiency of overexpression and downregulation was detected by using qRT-PCR.

### Quantitative real-time polymerase chain reaction (qRT-PCR) analysis

Total RNAs were extracted using Trizol reagents (Thermo, USA) according to the manufacturer’s protocol. The quantity and quality of total RNA samples were determined by a NanoDrop machine (DE, USA). Then, 0.5 μg of RNA extracted from clinical tissues or cells was subjected to reverse transcription and cDNAs were obtained (Applied Biosystems, USA) according to the manufacturer’s instructions. To detect the expression of miR-328-3p in the OS tissues and OS cell lines, qRT-PCR analysis was conducted using SYBR Green qPCR Master Mix (Roche, Switzerland). The reaction was conducted by using 1 μL cDNA, 10 μL SYBR Green qPCR Master Mix, 2 μL primers and 7 μL ddH_2_O, with a total volume of 20 μL. The conditions were as follows: 95 °C for 30 s, followed by 40 cycles of amplification at 95 °C for 5 s, at 59 °C for 30 s and at 72 °C for 30 s. The primer sequences used in our study were as follows: miR-328-3p forward, 5′-CGGGCCTGGCCCTCTCTGCC-3′ and reverse, 5′-CAGCCACAAAAGAGCACAAT-3′; U6 forward, 5′-CTCGCTTCGGCAGCACA-3′ and reverse, 5′-AACGCTTCACGAATTTGCGT-3′. The expression of miR-328-3p was normalized to that of U6 small nuclear RNA. The expression of miR-328-3p was quantified using the 2^−ΔΔCT^ method. ΔC_T_ = C_T_ (miR-328-3p)-C_T_ (U6). C_T_ was threshold cycle number.

### 3-(4,5-Dimethylthiazol-2-yl)-2,5-diphenyltetrazolium bromide (MTT) assay

To analyze the effect of miR-328-3p in the OS cells, the number of viable cells was determined by MTT assay according to the manufacturer’s protocols. In brief, the cells were seeded in a 96-well plate at a density of 1 × 10^5^ cells/well and cultured until the confluence reached about 80%. Then, the cells were stained with 10 μL MTT solutions (5 mg/mL, Biosharp, China) and incubated for 4 h at 37 °C until the formazan crystals were formed. After 4 h, the supernatant was removed and changed by 100 μL of dimethylsulfoxide (DMSO, Sigma-Aldrich, USA). After the mixture was agitated at a low speed for 10 min, the formazan crystals were dissolved in DMSO. Subsequently, the absorbance (at 490 nm) was detected by using a microtiter plate reader (TECAN, USA).

### Colony formation assay

MG-63 cells at a density of 1 × 10^3^ cells/well were cultured in 6-well plates, transfected with miRNAs and kept in culture medium for 2 weeks. The medium was changed every 3 days. The cells were fixed in the presence or absence of 4% paraformaldehyde for 20 min and the colonies were stained with 0.1% crystal violet (Biosharp, China) at room temperature. After 20 min, the cells were washed by using PBS for three times. At last, the number of colonies was analyzed and monitored using equation more than 50 cells/colony. An Olympus light microscope (Nikon, Japan) was used to capture images.

### Wound healing assay

The wound healing assay was used to assess the migration of OS cells. MG-63 cells were seeded in 6-well plates and the MG-63 cells grew to 80–90% confluence. Then, the cells were used for wound healing assay 24-h post-transfection. After the medium was removed, the cells were manually scraped by using clean sterile pipette tip and an artificial wound was obtained. Then, the cells were washed using phosphate-buffered saline (PBS, Solarbio, China) three times to remove cell debris, and the fresh medium was changed. Finally, the images were captured by using a microscope. The width of the wounding scratches at 0 h, 12 h, 24 h and 48 h was observed and captured. The percentage of wound healing was calculated using ImageJ software.

### Xenograft in vivo mice assay

The animal experiments were carried out in accordance with the Guide for the Care and Use of Laboratory Animals of the National Institutes of Health and were approved by the Animal Care and Use Committee of the First Affiliated Hospital of Harbin Medical University. For subcutaneous xenograft research, 32 five-week-old male Balb/c nude mice (20–25 g) were provided by the Beijing Vital River Laboratory Animal Technology, China and maintained under sterile specific-pathogen free (SPF) conditions. 3 × 10^6^ MG-63 cells transfected with miR-328-3p were trypsinized and injected subcutaneously into the flanks of the mice to determine the roles of miR-328-3p in the tumor growth. After 4 weeks, the mice were euthanized by anesthesia and the subcutaneous formatted tumor nodes were collected for further analysis. Then, the weights of tumor were measured and analyzed. Tumor growth was assessed via measuring the tumor volume and tumor weight.

### Analysis of cell apoptosis

The MG-63 cells were transfected with miR-328-3p mimic, miR-328-3p inhibitor and their corresponding NCs. The apoptosis of MG-63 cells was analyzed by using TUNEL staining according to the manufacturer’s protocol. Briefly, following transfection for 24 h, the cells were harvested and rinsed by PBS and then fixed using 4% PFA for 30 min at room temperature. After that, the cells were incubated by terminal deoxynucleotidyl transferase-mediated dUTP nickend labeling assay kit (Roche, Switzerland) according to the manufacturer’s instruction. Finally, the apoptotic cells were observed and captured under a microscope (Nikon, Japan). The apoptotic cells exhibited green color and the nucleus of cells showed blue color. Triplicate individual experiments were performed in this study.

### Target prediction

Bioinformatic analysis was conducted using TargetScan (http://www.targetscan.org/vert_71/), one online program that can predict targets of miRNAs by seeking the specific sequence complementary to the seed region of each miRNA. The analysis was used to explore the correlation between miR-328-3p and MMP-16 and verify whether MMP-16 3′-UTR contains the seed region of miR-328-3p.

### Western blot

To determine the expression of protein in cells, western blot was performed. First, ice-cold PBS was applied to wash the cells and total proteins were extracted from MG-63 cells with a radioimmunoprecipitation assay buffer (Thermo, USA) containing 0.5% SDS and 3% proteinase inhibitor cocktail for 30 min at 4 °C. The cell lysis was centrifuged at 12500 rpm/min for 10 min at 4 °C and the supernatants were collected. The proteins were qualified by BCA detecting kit (Beyotime, China) following the manufacturer’s protocols. Subsequently, the proteins were separated by sodium dodecyl sulfate polyacrylamide gel electrophoresis (SDS-PAGE) and transferred onto polyvinylidene difluoride (PVDF) membranes (Millipore, USA). After that, the membrane was blocked by bovine serum albumin and incubated with primary antibodies at 4 °C overnight. The next day, the membrane was incubated with secondary antibodies at room temperature for 1 h. The primary antibodies used in this study included: anti-β-actin antibody and anti-MMP-16 antibody from Cell Signaling Technology and anti-rabbit secondary antibodies were from Abcam. The protein expression level of β-actin was used as loading control. Protein expression levels were analyzed by Image Lab software (Bio-Rad Laboratories, USA).

### Luciferase reporter gene assay

The online database predicts that miR‑328-3p directly targets MMP-16. Relative luciferase activity was determined by using a Dual-Luciferase Reporter Assay System (Promega, USA) according to the manufacturer’s protocol. In brief, HEK293T cells at a density of 2 × 10^4^/cell were cultured in 24-well plates overnight. The 3′UTR of the human MMP-16 with the predicted miR-328-3p binding site was amplified and cloned to a psiCHECK vector. A combination of luciferase vectors carrying wildtype (WT) 3′-UTR or mutated 3′-UTR of MMP-16 were co-transfected with miR-328-3p mimic/miR-328-3p inhibitor into the cells using Lipofectamine 2000 reagents (Invitrogen, USA). After 48 h of transfection, the cells were collected and luciferase values were determined by using luciferase reporter assay kits. Subsequently, firefly luciferase activities were normalized to renilla luciferase activities.

### Statistical analysis

All data are presented as the mean ± S.E.M., and all the experiments were performed in triplicate. Differences between different groups were analyzed by Student’s test or One-way ANOVA. Statistical analyses in this study were finished using the Graphpad software. *P *< 0.05 was considered to indicate a statistically significant difference.

## Results

### MiR-328-3p is decreased both in OS tissues and cell lines

**Fig. 1:**
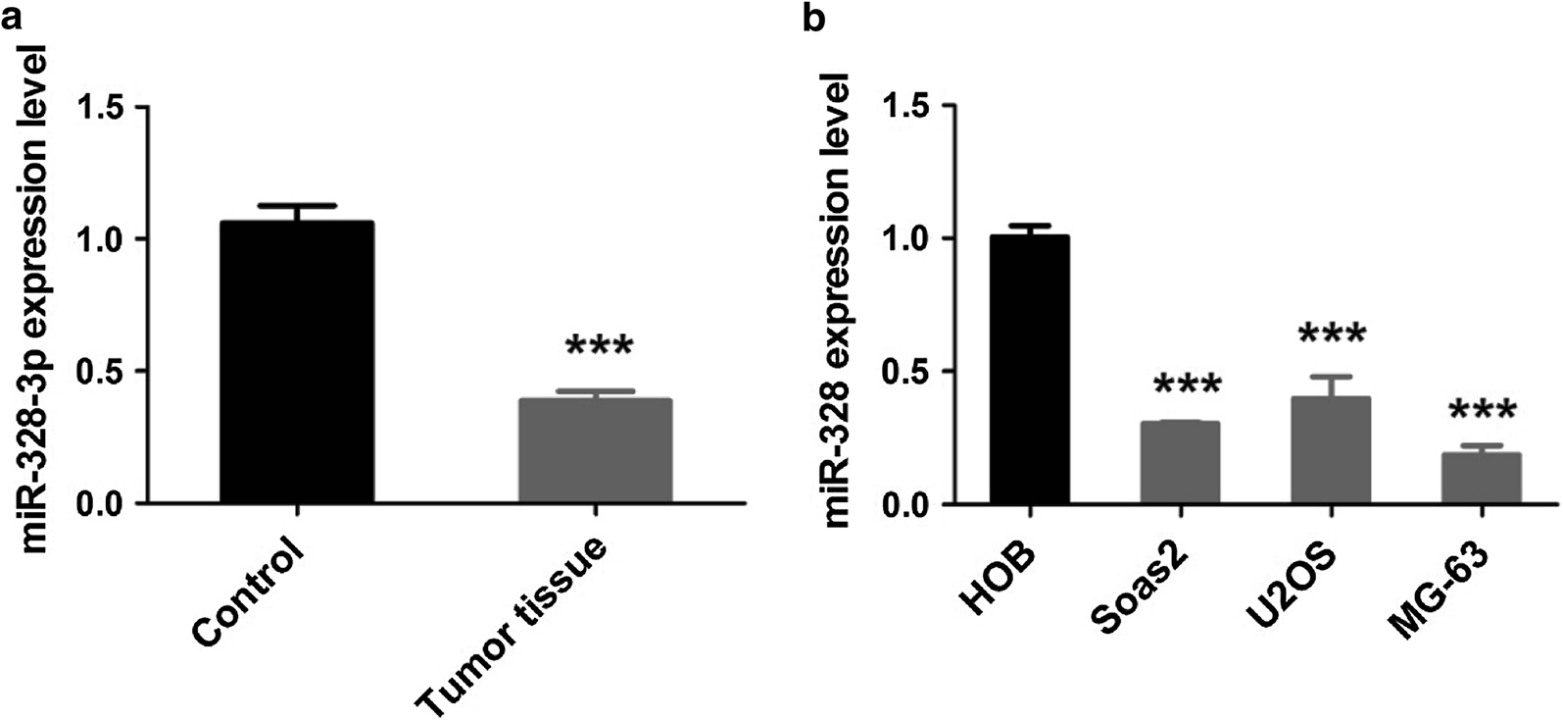
The expression of miR-328-3p in the OS tissues and cells. **a** Differentially expressed of miR-328-3p in OS tissues and corresponding adjacent tissues was verified by qRT-PCR. **b** The expression of miR-328-3p in the OS cells was detected by qRT-PCR analysis. ***p < 0.001 compared with control

To validate the role of miR-328-3p in OS, the expression levels of miR-328-3p in OS and cell lines were detected using qRT-PCR. First, we detected the expression of miR-328-3p in OS tissues and corresponding adjacent tissues (n = 3) by qRT-PCR. The results indicated that the expression of miR-328-3p was markedly lower in OS tissues than that in corresponding adjacent tissues (Fig. 1a). As shown in Fig. 1b, decreased levels of miR-328-3p were detected in OS cell lines in comparison with HOB cells (Fig. 1b). Among these cells, the expression of miR-328-3p was the lowest in MG-63 cell line. Therefore, MG-63 cells were chosen for further analysis.

### MiR-328-3p suppresses the cell growth and proliferation of OS cells

**Fig. 2:**
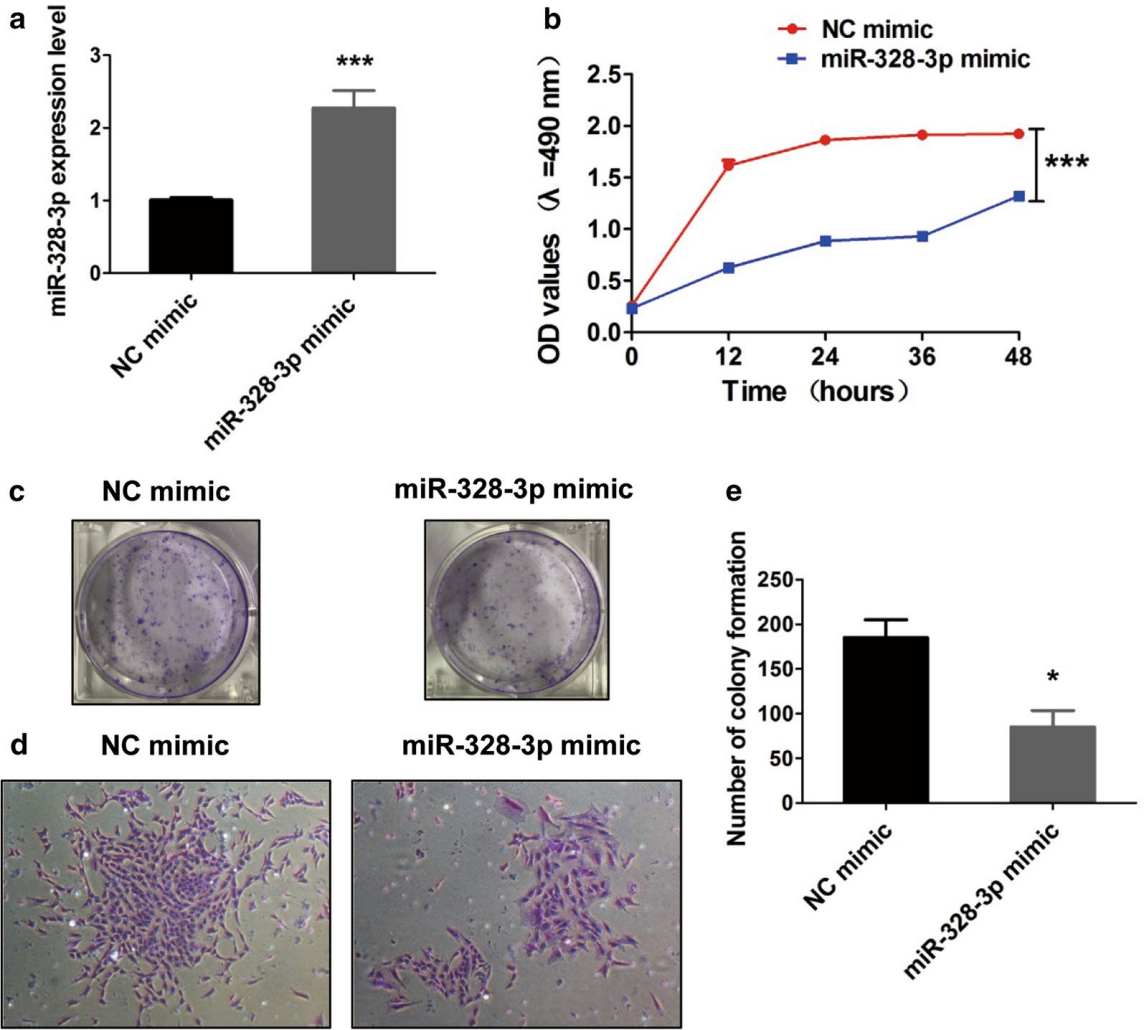
The effects of miR-328-3p mimic in the proliferation of OS cells. **a** The efficiency of miR-328-3p mimic in MG-63 cells. **b** Proliferation ability of MG-63 cells after transfection of miR-328-3p at different times. **c** The colony formation assay was used to detect the proliferation of MG-63 cells transfected with miR-328-3p. **d** The cell colonies were observed and captured following transfection with miR-328-3p. **e** The number of cell colonies was counted and analyzed. *p < 0.05 and ***p < 0.001 compared with control

To examine the biological effects of miR-328-3p in MG-63 cells, the cells were transfected with miR-328-3p mimic and NC. The results showed that the expression of miR-328-3p was observably increased after transfection of miR-328-3p mimic and the cells could be used for a follow-up experiment (Fig. 2a). Then, the cell growth of MG-63 cells was measured after transfection of 0 h, 12 h, 24 h, 36 h and 48 h. MTT assays proved that overexpression of miR-328-3p could obviously inhibit the proliferation of OS cells (Fig. 2b). Additionally, colony formation assay demonstrated a significant decrease of the number of colonies in MG-63 cells, suggesting that the proliferation of OS cells transfected with miR-328-3p was significantly inhibited (Fig. 2c, d). The statistical results were in accordance with the results (Fig. 2e). Collectively, these results indicated that overexpression of miR-328-3p inhibited the cell proliferation of MG-63 cells.

### MiR-328-3p promotes the apoptosis but inhibits the migration and tumor formation of OS cells

**Fig. 3:**
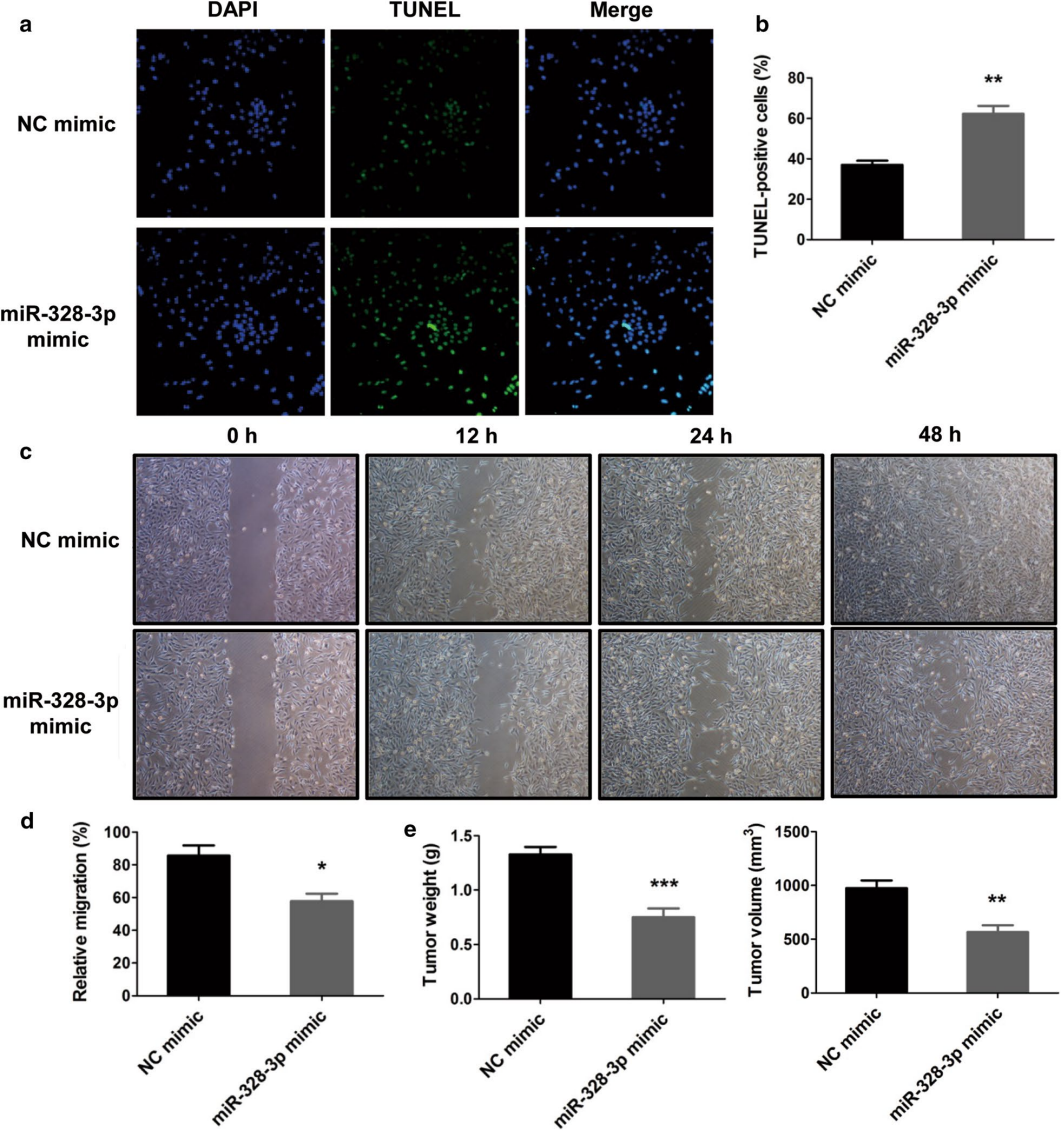
The effects of miR-328-3p mimic in the apoptosis, migration and tumor formation of OS cells. **a** TUNEL staining was performed to detect the apoptotic cells after treatment with miR-328-3p. **b** The number of TUNEL-positive cells was analyzed. **c** Cell scratch test after cell transfection with miR-328-3p mimic and NC mimic. **d** The migration ability of MG-63 cells was analyzed. **e** The tumor formation in vivo was assessed by subcutaneous tumor assay. *p < 0.05, **p < 0.01 and ***p < 0.001 compared with control

To further analyze whether miR-328-3p palyed a negative role in OS, the effects of miR-328-3p on the apoptosis, migration and tumor formation of OS cells were determined. Initially, the effect of miR-328-3p on the apoptosis of OS cells was examined by TUNEL staining. As the results presented in Fig. 3a, b, the number of apoptotic cells were elevated by transfection of miR-328-3p mimic, indicating that miR-328-3p could notably promote the apoptosis of OS cells, as compared to NC mimic. Furthermore, the wound healing assay was performed to detect the migration of MG-63 cells after transfection. As shown in Fig. 3c, d, the results revealed that the migration of MG-63 cells was significantly suppressed by overexpression of miR-328-3p. In addition, to explore the tumor-inhibitor role of miR-328-3p in OS cells, xenograft nude mice models, which were subcutaneously injected with MG-63 cells, which were transfected with miR-328-3p mimic, were established. The tumors were monitored and dissected 4 weeks after xenografting. The results uncovered that the tumor volume and weight of the MG-63 cells transfected with miR-328-3p mimic were visibly reduced relative to the control group (Fig. 3e). Therefore, we concluded that miR-328-3p could accelerate the apoptosis, but inhibit the migration and tumor growth of OS cells.

### Knockdown of miR-328-3p improves the cell growth and proliferation of OS cells

**Fig. 4:**
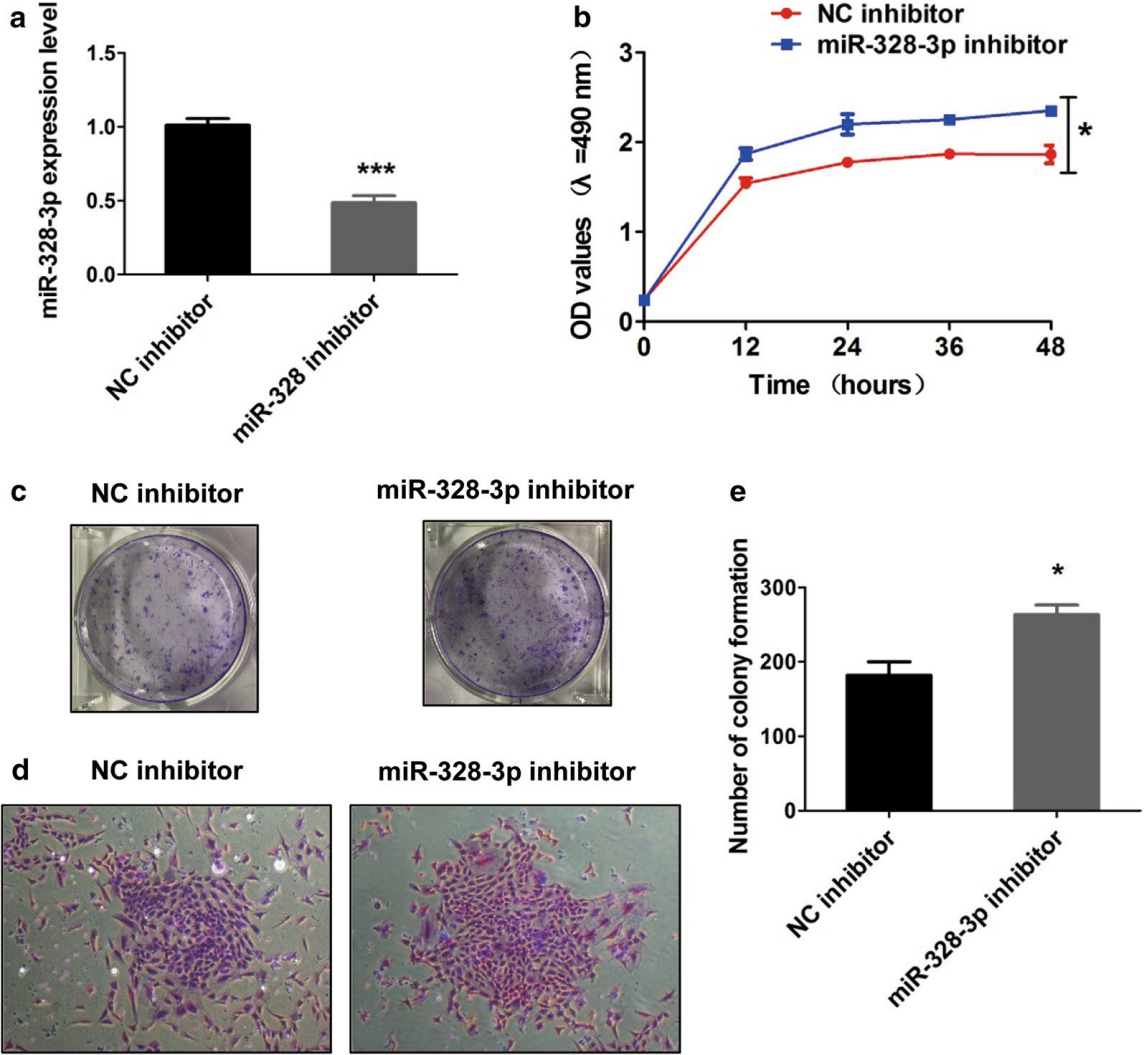
The roles of miR-328-3p inhibitor in the proliferation of OS cells. **a** The efficiency of downregulation was assessed by qRT-PCR assay. **b** MMT assay was used to detect the cell viability of MG-63 cells. **c**, **d** Knockdown of miR-328-3p led to increased proliferation capacity in MG-63 cells. **e** The relative number of colonies was increased by miR-328-3p inhibitor. *p < 0.05 and ***p < 0.001 compared with control

Having confirmed the negative correlation of miR-328-3p with the cell growth and proliferation in OS cells, we explored the functions of miR-328-3p inhibitor in OS cells. We found that miR-328-3p was obviously reduced in MG-63 cells after transfection of miR-328-3p inhibitor relative to NC inhibitor, so the cells were selected for subsequent studies (Fig. 4a, b). Next, MTT assay revealed that decrease of miR-328 significantly promoted the cell growth of MG-63 cells (Fig. 4c). Moreover, knockdown of miR-328-3p significantly increased the proliferation ability of MG-63 cell lines (Fig. 4e). Taken together, miR-328-3p inhibitor promoted the cell growth and proliferation of OS cells, which was in contrast with miR-328-3p.

### Downregulation of miR-328-3p decreases the apoptosis but increases the migration and tumor formation of OS cells

**Fig. 5:**
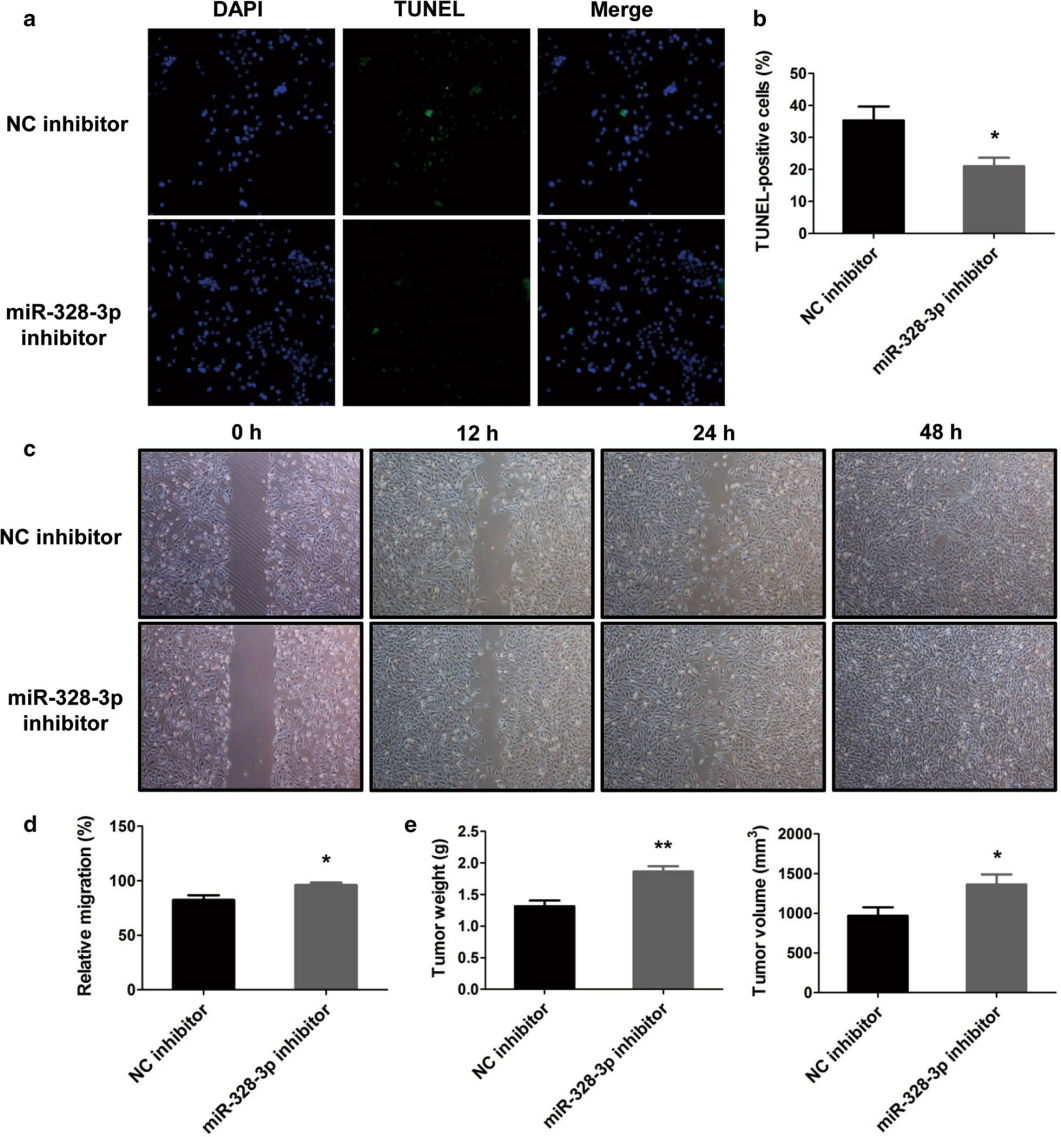
The roles of miR-328-3p inhibitor in the apoptosis, migration and tumor formation of OS cells. **a**, **b** Decreased miR-328-3p expression inhibited the apoptosis of MG-63 cells. **c**, **d** The relative migrated cells were shown after transfection of miR-328-3p inhibitor. **e** Xenograft in vivo mice assays were performed to test the influence of miR-328-3p inhibitor on the tumor formation of MG-63 cells. *p < 0.05, **p < 0.01 compared with control

To further ascertain whether downregulation of miR-328-3p played a role in OS cells, the MG-63 cells were transfected with miR-328-3p inhibitor to silence the expression of miR-328-3p. Cell apoptosis was detected using TUNEL staining. And knockdown of miR-328-3p decreased the TUNEL-positive cells, suggesting miR-328-3p inhibitor inhibited the apoptosis of MG-63 cells (Fig. 5a, b). As depicted in Fig. 5c, d, miR-328-3p inhibitor significantly promoted the migration of MG-63 cells. To further explore the effects of miR-328-3p inhibitor on the tumor growth, the animal experiments were carried out. A subcutaneous tumor assay was performed, and the tumor size was measured regularly. As expected, the tumor formation experiments in vivo indicated that the tumor growth was elevated by knockdown of miR-328-3p (Fig. 5e). Taken together, we concluded that downregulation of miR-328-3p was able to decrease the apoptosis, but increase the migration and tumor growth of OS cells.

### MiR-328-3p negatively regulates the OS cells through inhibiting MMP-16

**Fig. 6:**
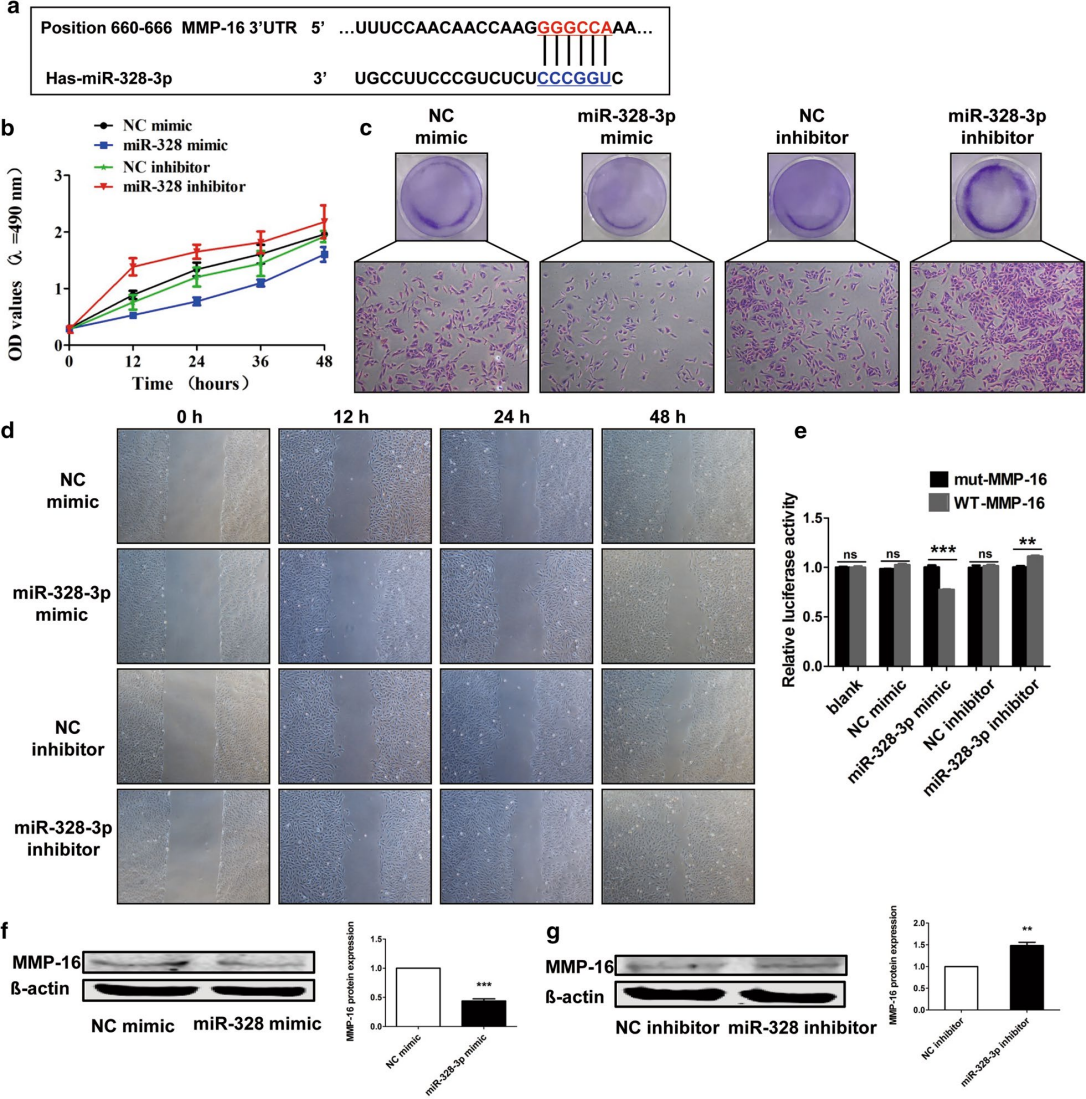
MMP-16 is a potential target of miR-328-3p in OS cells. **a** Predicted miR-328-3p target sequence in the 3′-UTR of MMP-16 was shown. **b** The role of mi-328-3p and MMP-16 in the cell viability of MG-63 cells. **c** The proliferation of MG-63 cells was inhibited by miR-328-3p but elevated by MMP-16. **d** The effects of miR-328-3p and MMP-16 on the migration of MG-63 cells. **e** Analysis of relative luciferase activities of MMP-16-WT and MMP-16-mut. **f**, **g** Western blot analysis of MMP-16 in MG-63 cells after transfection of miR-328-3p mimic and inhibitor. **p < 0.01 and ***p < 0.001 compared with control

To gain insight into the molecular mechanisms by which target gene mediated miR-328-3p to regulate the progression of OS cells, the target genes were predicted by TargetScan databases online. As shown in Fig. 6a, MMP-16 is a target gene of miR-328-3p and the binding sites between miR-328-3p and MMP-16 were shown. MTT assay indicated that miR-328-3p decreased the cell viability of MG-63 cells, while overexpression of MMP-16 increased the cell viability in MG-63 cells (Fig. 6b). And co-transfection of miR-328-3p and MMP-16 reversed the increase in the cell viability caused by MMP-16 (Fig. 6b). In addition, colony formation assay revealed that miR-328-3p could decreased the proliferation of MG-63 cells, which was increased by overexpression of MMP-16 (Fig. 6c). As shown in Fig. 6d, the migration ability was inhibited by miR-328-3p, but elevated by MMP-16 (Fig. 6d). Luciferase reporter assay was used to determine whether MMP-16 was regulated by miR-328-3p. The results suggested that the relative activity of luciferase in the reporter which contained a wild-type MMP-16 3′-UTR was cut down in the presence of miR-328-3p mimic (Fig. 6e). As a further confirmation of the above findings, the expression of MMP-16 in MG-63 cells after transfection of miR-328-3p was examined. Western blot analysis showed that overexpression of miR-328-3p markedly decreased the protein expression levels of MMP-16, indicating that miR-328-3p negatively regulated MMP-16 expression in OS cells (Fig. 6f). Moreover, the results also indicated that knockdown of miR-328-3p significantly enhanced the expression of MMP-16 (Fig. 6g). These findings indicated that miR-328-3p mediated the anti-tumor effect in OS by targeting MMP-16.

## Discussion

MiRNAs are a type of evolutionarily conserved noncoding RNAs which are about 18–25 nt in length and negatively regulate the gene expression at the posttranscriptional level by directly inhibiting the target genes [22, 23]. The biological function and mechanism of miRNAs have been studied broadly.

In recent, accumulating evidence demonstrated that several miRNAs were dysregulated in types of tumors, and the oncogenic or anti-cancer roles of miRNA were obvious in various cancers [24–26]. For example, miR-125b has been found to play a vital role in the development of malignant tumors [3]. MiR-590 regulated the chemoresistance of osteosarcoma by targeting wild-type p53-induced phosphatase 1 (WIP1) [27]. Besides, miR-1301 was reported to be an inhibitor of tumorigenesis in hepatocellular carcinoma cells [28]. In addition, there were some reports indicated that miRNAs could regulate the progression and developments of osteosarcoma (OS) [23, 29, 30]. In the previous report, we found that miR-328-3p was significantly decreased in the hepatocellular carcinoma (HCC) tissues, which was validated by gene microarray analysis [31]. However, there was no reports have shown that miR-328-3p is related to the development of OS.

In presents study, the data indicated that miR-328-3p was significantly decreased both in OS tissues and cell lines. And overexpression of miR-328-3p inhibited the cell growth and proliferation of MG-63 cells. Additional analysis showed that miR-328-3p could decrease the migration and tumor formation, but promote the apoptosis of MG-63 cells. More importantly, knockdown of miR-328-3p exhibited the opposite effects compared with overexpression of miR-328-3p. In the presents study, we first studied miR-328-3p expression in the OS tissues and OS cells, and found miR-328-3p might be a vital target in treating OS. Functional study demonstrated that overexpression of miR-328-3p inhibited the development of OS.

In our study, further experiments suggested that miR-328-3p regulated the development of OS by targeting matrix metalloproteinase-16 (MMP-16). MMP-16 is a member of the matrix metalloproteinase (MMP) family of proteins [32]. It has been reported that MMP proteins are related to extracellular matrix breakdown in normal physiological processes such as embryonic development, reproduction, tissue remodeling, and in physical disease processes [33–35]. For example, MMP-2 and MMP-9 have been reported to play a key role in the invasive of various tumors through degrading the basement membrane. MMP-9 can participate in capsular infiltration in hepatocellular carcinoma (HCC) [36]. According to the reports, MMP-16 promotes the migration and invasion of gastric cancer (GC) cells and thus causes worse survival outcome in GC [37]. In addition, it has been reported that miR-328-3p enhanced the radiosensitivity, inhibited the proliferation and promoted apoptosis in osteosarcoma cells under radiation conditions by directly targeting H2AX [38]. In our study, miR-328-3p can inhibit the cell growth, proliferation and promote the apoptosis of OS cells, which was in consistent with the previous reports. However, we further detected the role of miR-328-3p in the migration and tumor growth of OS cells and found that miR-328-3p regulated the development of OS by downregulating the expression of MMP-16. Thus, miR-328-3p mediates the anti-tumor effect in osteosarcoma via directly targeting MMP-16.

## Conclusion

This study demonstrated that miR-328-3p can significantly suppress the proliferation, migration and tumor formation, but induce the apoptosis of osteosarcoma cells by directly targeting MMP-16. MiR-328-3p plays a critical role in the development and occurrence of OS. The present study may provide a novel target for intervention, diagnosis and treatment of OS. Overexpression of miR-328-3p in OS represents an attractive target in the treatment of OS in clinic.

## Abbreviations

miRNAs: microRNAs
OS: osteosarcoma
MMP-16: matrix metalloproteinase-16
3′-UTR: 3′-untranslated region
mRNAs: messenger RNAs
HOB: human osteoblasts
DMEM: Dulbecco’s modified Eagle’s medium
qRT-PCR: quantitative real-time polymerase chain reaction
MTT: 3-(4,5-dimethylthiazol-2-yl)-2,5-diphenyltetrazolium bromide
DMSO: dimethylsulfoxide
PBS: phosphate-buffered saline
SPF: specific-pathogen free
SDS-PAGE: sodium dodecyl sulfate polyacrylamide gel electrophoresis
PVDF: polyvinylidene difluoride
WT: wildtype
HCC: hepatocellular carcinoma
MMP: matrix metalloproteinase
GC: gastric cancer
